# Human papillomavirus in Italy: retrospective cohort analysis and preliminary vaccination effect from real-world data

**DOI:** 10.1007/s10198-021-01317-w

**Published:** 2021-06-12

**Authors:** A. Marcellusi, F. S. Mennini, P. Sciattella, G. Favato

**Affiliations:** 1 Economic Evaluation and HTA (EEHTA), CEIS, Faculty of Economics, University of Rome “Tor Vergata”, Rome, Italy; 2 Institute for Leadership and Management in Health, Kingston University London, London, UK

**Keywords:** Human papillomavirus, Real-world data, Hospitalization, Public health prevention, I18, Government policy, Regulation, Public health

## Abstract

**Introduction:**

The objective of this study was to estimate the lifetime risk of hospitalization associated with all major human papillomavirus (HPV)-related diseases in Italy. Moreover, a preliminary vaccination effect was also performed.

**Methods:**

A retrospective, nonrandomized, observational study was developed based on patients hospitalized between 2006 and 2018 in Italy. All hospitalizations were identified through administrative archives, according to the International Classification of Diseases (ICD-9 CM). Information related to the hospital discharges of all accredited public and private hospitals, both for ordinary and day care regimes, was taken into account. We included hospitalizations related to resident patients presenting one of the ICD-9-CM codes as primary or secondary diagnosis: genital warts (GW); ‘cervical intraepithelial neoplasia (CIN)’ (067.32–067.33); ‘condyloma acuminatum’ (078.11); ‘anal cancers’ (AC) (154.2–154.8); oropharyngeal cancers (OC): ‘oropharyngeal cancer’(146.0–146.9) and ‘head, face and neck cancers’ (171.0); genital cancers (GC): ‘penis cancer’ (187.1–187.9) and ‘cervical cancer’ (180.0–180.9). Data were stratified by birth year and divided into two groups: (a) cohort born before 1996 (not vaccinable) and (b) cohort born after 1997 (vaccinable—first cohort that could be vaccinated at the beginning of immunization schedule in girls since 2008 in Italy). Disease-specific hospitalization risks for both groups were estimated by sex, year and age.

**Results:**

Epidemiological data demonstrate that the peak hospitalization risk occurred at 24–26 years of age for GW (both male and female); 33–41 and 47–54 years for AC males and females, respectively; 53–59 and 52–58 years for OC males and females, respectively; and 54–60 and 39–46 years for GC males and females, respectively. Focusing on GW and GC, vaccinable females demonstrate a significant reduction in hospitalization risks (− 54% on average) compared to nonvaccinable females until 21 years of age (maximum follow-up available for girls born after 1997). Comparing the same birth cohort of males, no differences in hospitalization risk were found.

**Conclusions:**

These results support the importance of primary prevention strategies in Italy and suggest that increased VCRs and time of observation (genital cancers for which vaccination is highly effective, have a latency of some decades) will provide useful information for decision-makers.

## Introduction

Human papillomavirus (HPV) is the most common sexually transmitted virus and causes a substantial burden of disease in both men and women [1]. In 2013–2014, approximately 45% of men and 40% of women between the ages of 18 and 59 had genital HPV infection [2], a large proportion of which is preventable with vaccination.

Since the first prophylactic vaccine against Human Papillomavirus (HPV) was licensed in 2006, the quadrivalent vaccine (which protects against high-risk HPV types 16 and 18, and low-risk types 6 and 11, which cause 90% of genital warts) or bivalent vaccine (targeting HPV types 16 and 18) have been implemented in more than 28 countries as part of their national immunization programmes [3]. In December 2014, a nonavalent vaccine (9vHPV), developed r. The 9vHPV gained marketing authorisation from the European Commission, valid throughout the European Union, in June 2015.

The HPV vaccine has been shown to be close to 100% effective against benign external anogenital warts (GWs) [4] and against persistent infection with HPV16 and 18 [5], which are responsible for 70% of all cervical cancers [6]. HPV also accounts for a smaller fraction of cancers of the vulva, vagina, anus, penis, head, and neck [7]. The low-risk HPV variants of genotypes 6 and 11 are responsible for approximately 90% of benign external anogenital warts [8] and almost all cases of recurrent respiratory papillomatosis (RRP) [9, 10].

At the beginning, the vaccination was mainly targeted at girls between the ages of 9 and 12 years, before the onset of sexual activity by females; however, various countries have also implemented gender neutral primary prevention strategies enrolling for boys at the same age [11].

Primary prevention of HPV-related diseases in Italy started in 2008, and the vaccination programme consists of active and free service for all 12-year-old girls. Moreover, in 2017, the Italian Ministry of Health extended the immunization programme to 12-year-old boys, even though some regions had already started in 2014 [12]. Although different studies were conducted in Italy to evaluate the epidemiological impact [13, 14], economic burden [15–17], and vaccination cost-effectiveness [11, 18–22] of HPV-related diseases, no specific analyses were conducted from a cohort real-world data perspective.

The objective of this study is to estimate the lifetime risk of hospitalization and direct costs associated with human papillomavirus (HPV)-related disease in Italy. Moreover, a preliminary vaccination effect was also performed.

## Methods

A retrospective, nonrandomised, observational study was developed based on patients hospitalized between 2006 and 2018 in Italy. All hospitalisations were identified through administrative archives, according to the International Classification of Diseases (ICD-9 CM). Information related to the hospital discharges of all accredited public and private hospitals, both for ordinary and day care regimes, was taken into account. We included hospitalisations related to resident patients presenting one of the ICD-9-CM codes as primary or secondary diagnosis: genital warts (GW): ‘condyloma acuminatum’ (078.11); ‘cervical intraepithelial neoplasia (CIN)’ (067.32–067.33); ‘anal cancers’ (AC) (154.2–154.8); ‘oropharyngeal cancers (OC): ‘oropharyngeal cancer’ (146.0–146.9) and ‘head, face and neck cancers’ (171.0); and genital cancers (GC): ‘penis cancer’ (187.1–187.9) and ‘cervical cancer’ (180.0–180.9) [16, 17]. A sensitivity analysis was applied considering only the primary diagnosis.

Overall hospitalization rates were calculated as the ratio between the number of hospitalisations at each age of the cohort and the number of residents in Italy with the same age and for the same cohort [23]. Data were then stratified by birth years, age of hospitalization, and ICD-9-CM group.

Given the highest incidence rate of GW and CIN among young people [24] and their short latency after HPV infection, a focus was made on this condition among females. The GW and CIN female cohort was divided into two groups: (a) a cohort born before 1996 (not vaccinable) and (b) a cohort born after 1997 (vaccinable—the first cohort that was offered vaccination starting from 2008). The contemporary rates were estimated by year, age, and sex for both hospitalization and patients. For other demographic analyses, the ideal aim of this work would represent a generation of individuals hospitalized through time in Italy and measure the probabilities of hospitalization and costs occurring in each generation at different ages until the whole cohort became extinct [25]. However, the limited number of years covered with national data (11 years for each cohort) cannot cover the entire lifespan of the cohort, and it is not possible to calculate the age-specific probabilities of hospitalization calculated using observed hospitalization (or patients hospitalized) data from the cohort. For this reason, a hypothetical cohort of 250,000 boys and girls was simulated, assuming that the hospitalization risk of a single generation at every age corresponds to the average risk estimated on different contemporary cohorts [26].

The total costs related to hospitalisations were calculated using the DRGs (with 2013 values) of hospitalized patients based on their age, gender and consumption of resources during their hospital stay. Information related to the hospital discharges (HDRs) of all accredited public and private hospitals in Italy, both for ordinary and day care regimens, was included to estimate the costs of cervical, vulvar, vaginal, penile, head, and neck cancer. Hospitalization and lifetime costs for these HPV-related conditions were investigated in-depth in previous work [17].

To combine real-world data and the analysis of the two hypothetical cohorts, the overall hospitalization rates for each disease were multiplied to a hypothetical 250,000 women and 250,000 men lived at each age of the cohort. The number of hospitalization by age was multiplied by the average hospitalization costs for each age estimated by the DRGs tariff. Finally, a simple forecast analysis for CIN and GW by age was simulated considering the average observed reduction of hospitalization rates for vaccinable cohort vs not vaccinable patients between 17 and 21 years old. Cost and risk reduction were estimated as absolute and percentage difference, respectively.

## Results

Our results covered 3 areas: (i) hospitalization rates and cost by life birth, sex, and HPV-related condition; (ii) hospitalization rate reduction per GW among women vaccinated and not vaccinated; and (iii) lifetime direct cost savings given the observed drop in hospitalization rate for GWs.

**Fig. 1:**
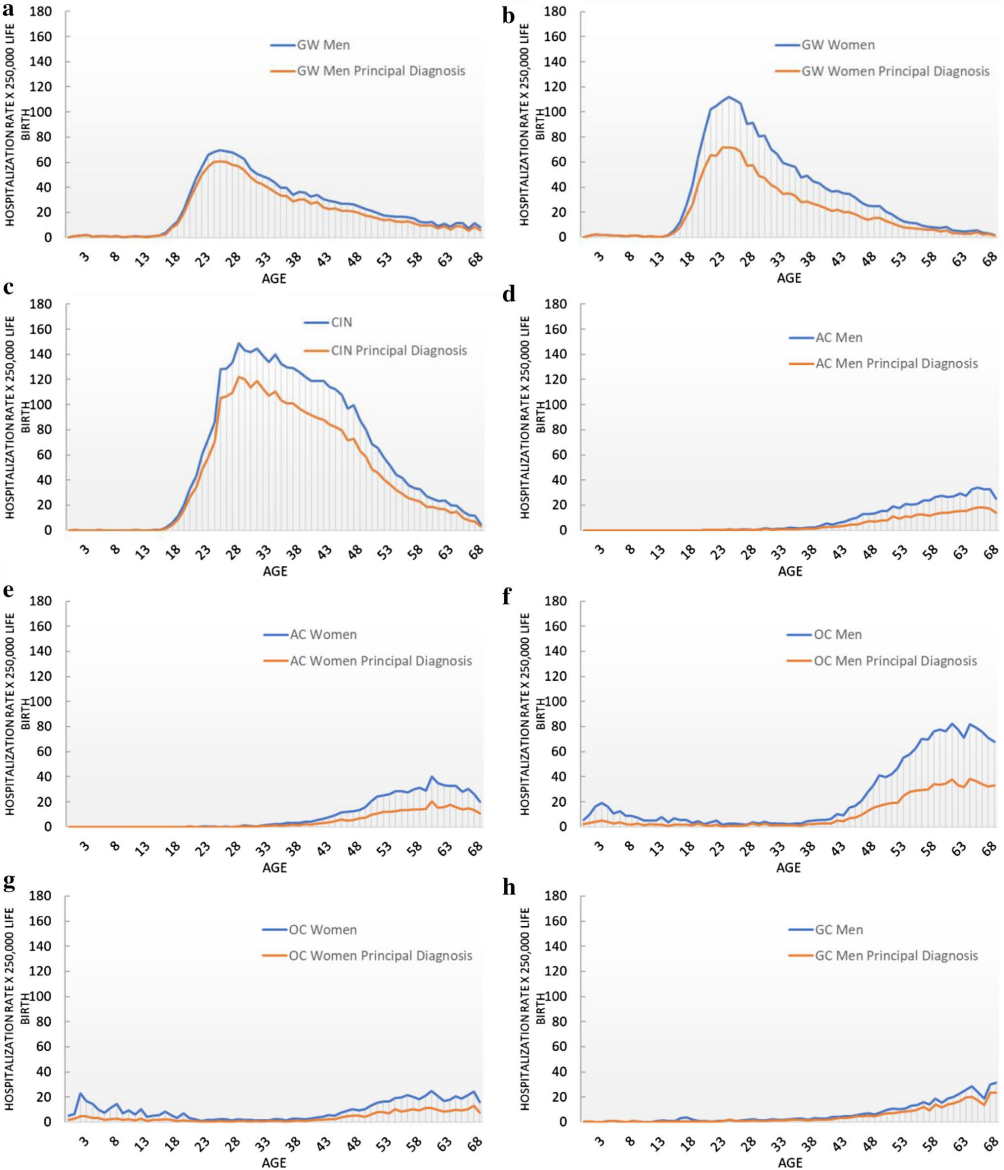

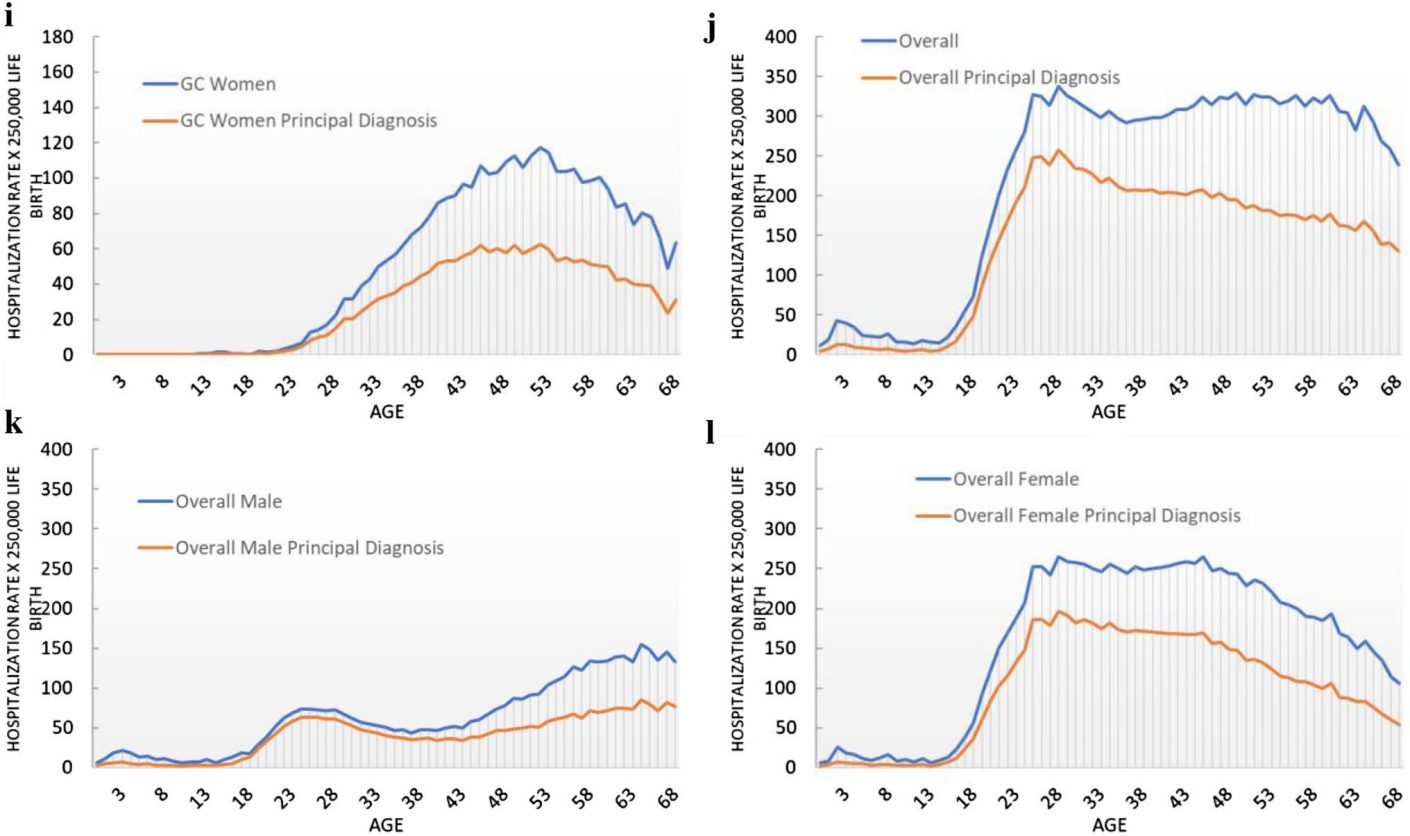
Hospitalization rate by age per 250,000 live births (primary or secondary diagnosis and primary diagnosis only). *GW* genital warts, *CIN* cervical intraepithelial neoplasia, *AC* anal cancer, *OC* oropharyngeal cancer, *GC* genital cancer

Figure 1 shows the hospitalization rate by age of a hypothetical cohort of 250,000 girls or boys born, close to the yearly natality observed in Italy. CIN, GW, and GC hospitalization rates are most frequent for women, with peaks between 20 and 27 and 48 and 58 years of age, respectively. A higher risk for men was registered for OC, with a peak between 58 and 68 years. Similar risks for both sexes were registered for AC. If we considered principal diagnosis as inclusion criteria, on average 81%, 68%, and 61% of the overall hospitalization were registered for GW in men, GW women, and CIN, respectively (orange line of Fig. 1). A lower percentage of principal diagnosis were registered for cancers (46%, 41%, and 56% on average for AC, OC, and GC, respectively).

A total lifetime cost of € 51.4 million was estimated for hospitalization cost in Italy (67% of which related to OC and GC—Table 1 section a). A total of 66% of the overall hospitalization costs are associated with women’s diseases (€ 33.9 million), and the most relevant economic burden was attributed to GC for women (54% of the total women hospitalization cost) and OC for men (58% of the total men hospitalization cost). Considering hospitalization with a primary diagnosis as the only inclusion criteria, the overall costs decrease to € 29.2 million (− 43% of the overall costs). In this secondary analysis, the overall costs associated with women decrease to 59%, while the overall distribution of the economic burden between the diseases remain constant (Table 2 section b).

**Table 1:** Lifetime hospitalization cost per 250,000 live births distributed by inclusion criteria classification

	Women	Men	Total
a. Primary or secondary diagnosis inclusion criteria			
Genital warts	€ 2.772.884	€ 1.909.803	€ 4.682.687
CIN	€ 5.311.117		€ 5.311.117
Anal cancer	€ 3.595.604	€ 3.515.654	€ 7.111.257
Oropharyngeal cancer	€ 3.947.230	€ 10.149.871	€ 14.097.101
Genital cancer	€ 18.267.346	€ 1.895.647	€ 20.162.993
Total	€ 33.894.181	€ 17.470.974	€ 51.365.155
b. Primary diagnosis inclusion criteria			
Genital warts	€ 720.770	€ 1.597.477	€ 2.318.247
CIN	€ 3.264.413		€ 3.264.413
Anal cancer	€ 1.724.001	€ 1.856.338	€ 3.580.340
Oropharyngeal cancer	€ 1.584.460	€ 6.908.219	€ 8.492.678
Genital cancer	€ 10.133.276	€ 1.366.860	€ 11.500.136
Total	€ 17.426.920	€ 11.728.893	€ 29.155.814

**Table 2:** Hospitalization cost per 250,000 live births

	Observed cost 10—21 years	Lifetime cost
GW women overall	€ 432,497	€ 2,772,884
GW women not vaccinated	€ 495,729	€ 2,836,116
GW women vaccinated	€ 190,464	€ 1,154,654*
Not vaccinated—Vaccinated	€ 305,265	€ 1,681,462
	Observed cost 10—21 years	Lifetime cost
CIN women overall	€ 153,310	€ 5,311,117
CIN women not vaccinated	€ 173,414	€ 5,331,188
CIN women vaccinated	€ 60,197	€ 1,741,231*
Not vaccinated—Vaccinated	€ 113,217	€ 3,589,957
	Observed cost 10—21 years	Lifetime cost
GW/CIN women overall	€ 585,807	€ 8,084,001
GW/CIN women not vaccinated	€ 669,143	€ 8,167,304
GW/CIN women vaccinated	€ 250,661	€ 2,895,885*
Not vaccinated—vaccinated	€ 418,482	€ 5,271,419

*GW* genital warts, *CIN* cervical intraepithelial neoplasia, *AC* anal cancer, *OC* oropharyngeal cancer, *GC* genital cancer

*Assuming the same average risk reduction for all ages after 21 years

**Fig. 2:**
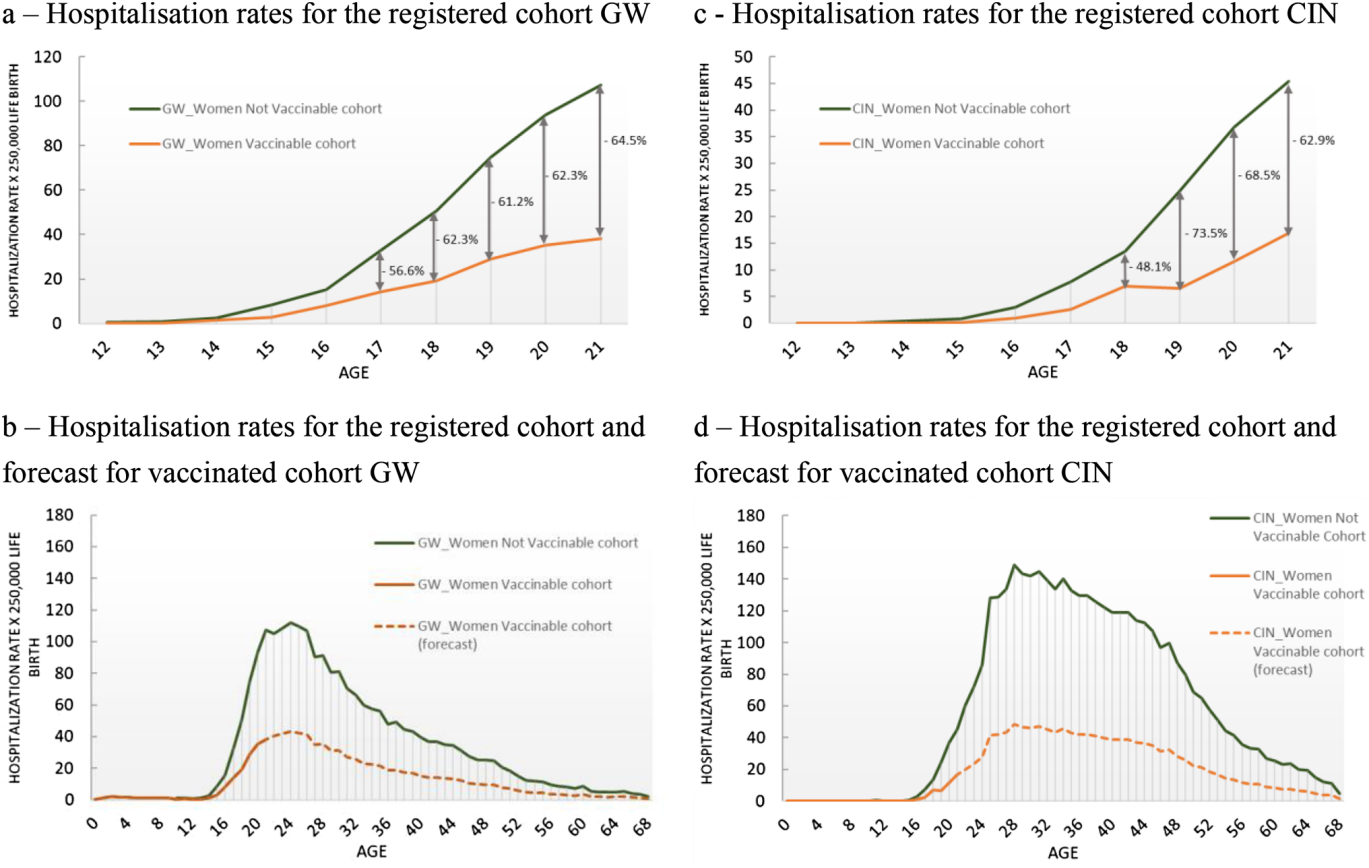
Hospitalization rate per 250,000 live births—GW women vaccinated and not vaccinated cohort. *GW* genital warts, *CIN* cervical intraepithelial neoplasia

Focusing on GW and CIN for women, Fig. 2 shows the hospitalization risks for two hypothetical cohorts of 250,000 women who follow the rate registered for the women (a) born before 1996 (not vaccinable) and (b) born after 1997 (vaccinable—the first cohort that was offered vaccination starting from 2008). The vaccinable cohort registered lower hospitalization rates for all comparable ages, with an average reduction of 61% and 67% between 17 and 21 years old for GW and CIN, respectively (Fig. 2a). Figure 2b, instead, shows the observed hospitalization age-specific rates for the not vaccinable cohort and the hypothetical risk reduction for vaccinated women after 21 years (the last age in which vaccination can be assumed), projecting constant average risk reduction over the lifetime span.

GW observed hospitalization cost reduction for the vaccinable cohort corresponds to − € 305,265 (− 62% of the overall cost) compared with not vaccinable cohort. These reductions increase to € 1.6 million if the same reduction rate is applied to the lifetime cost (Table 2).

## Discussion

The purpose of this study was to estimate the lifetime risk of hospitalization and direct costs associated with human papillomavirus (HPV)-related disease in Italy. Moreover, a preliminary vaccination effect was also performed.

Our study adopted a real-world data approach to estimate lifetime hospitalization risk and costs and conducted a very preliminary assumption on estimating the actual cost reduction attributable to the vaccination strategy in Italy considering the most recent data available. Additionally, we included a lifetime cost reduction scenario for GW in women, assuming that the observed risk and cost reduction remain constant over the observed ages in vaccinable women. Therefore, the present study was the first to measure the lifetime economic burden of HPV-related diseases in Italy considering a longitudinal perspective with newly available administrative data. By estimating the resource consumption attributable to the different cohorts, we aim to predict the effects of the strategies adopted in Italy at the beginning of vaccination introduction and to inform future public health decisions.

According to the results of this study, the hospitalization risk profile by age demonstrates the high impact of cancer condition for adult patients (two-thirds of the overall hospitalization occurs after 50 years), while GW and CIN hospitalization occurs for young adults (half of the total hospitalization before 38 years of the observed women). Furthermore, the economic burden among men represented more than one-third (34%) of the total hospitalization cost associated with the HPV-related diseases analyzed, which is consistent with previously published data [8, 17, 27] and with the effort to extend the anti-HPV immunization programme to include boys in the National Immunization Plan 2017–19.

Moreover, hospitalization rates were consistent with the epidemiological profile of women in Italy [13, 28–30], while the cost results represent, to our knowledge, the first and original data in the national and international literature. Indeed, this study estimated that considering a hypothetical cohort of 250,000 men and 250,000 women (on average, the number of live births in the last decade in Italy), we can expect a total hospitalization cost of € 51 million.

Analyzing GW and CIN for women, our study demonstrates that the hospitalization risks for women born before 1996 (not vaccinable) and after 1997 (vaccinable—the first cohort that was offered vaccination starting from 2008) were strongly different. The vaccinable cohort registered lower hospitalization rates for all comparable ages (− 61% and -67% between 17 and 21 years old for GW and CIN, respectively). These reductions demonstrate the first effect of the Italian public prevention strategy adopted in Italy against the HPV-related diseases during 2008–2012 [12].

In 2017, the Italian NHS had an average annual expenditure of € 38.3 million for the acquisition of the anti-HPV vaccine for the vaccination strategy between 2008 and 2018 [31]. If this investment leads to a hospitalization risk reduction for GC, CIN, and GW (similar association to HPV) in both sexes as estimated in this analysis, Italian NHS savings only for these hospitalisations cost conditions around € 19.7 million (38% of the total lifetime hospitalization cost and 51% of the vaccination prevention strategy investment). These savings could increase when the efficacy of the Hpv9 vaccination strategies are observable for AC and OC, demonstrating not only a very high impact in terms of morbidity and mortality [13] but also an efficient investment in terms of public health decisions [12, 18, 19].

The present study has several limitations. First, real-world data from administrative archives were only available for hospitalization at the national level, and published Italian sources of cost data were limited. The use of hospitalization data sources may have a high impact on the underestimation of the lifetime economic burden due to the lack of DRG tariff updates in the Italian context. Furthermore, no estimation was made in terms of drug, outpatient, and indirect costs. However, due to the increased number of outpatient management of these diseases (especially for GW) and the high impact of cancers on the productivity aspects of the diagnosed patients, we can assume that the economic burden estimated in this work represents just one-third of the total impact from the social perspective [12, 18, 27, 32–34].

A second limitation refers to the estimation of the vaccination effect on the GW for women. We can assume that the hospitalization reduction registered for vaccinable cohorts (born after 2017) in Italy is the combination of three main drivers: (1) time, (2) efficacy of the vaccine, and (3) vaccine active offer. The first variable reflects the changes in terms of management of the disease during the years considered (inpatient vs outpatient management). In Italy, the hospitalization rate has decreased for many years, and this is particularly true for the GW and CIN considered cases in younger ages (− 40% on average between the period 2006–2008 and 2016–2018). This difference was also expected for the management of GW for men. However, in this case, no difference was registered in terms of hospitalization reduction between the corresponding two cohorts of boys (born after or before 2017). Moreover, the hospitalization reduction is also a function of the efficacy of the vaccine that reflects the coverage rate of the different cohorts considered in the analysis and the mix of bivalent and quadrivalent vaccines used for these cohorts (bivalent vaccine has no immunization HPV 6 and 9 that are related to GW). Finally, age represents the variable that reflects the effect of primary preventive strategies. The vaccinable cohort registered -62% of hospitalization during the ages between 16 and 21, demonstrating the first indicative effects of the public health intervention proposed in Italy between 2006 and 2011.

These limitations should be considered in future research; however, in our opinion, they do not undermine the validity of the estimates in the present study or their estimated impact on the total lifetime economic burden of HPV-related diseases. Future research should address these gaps in epidemiological and cost data to reduce the uncertainty associated with the present estimates and evaluate the real effects for cancer diseases.

## Conclusion

This study represents the first economic analysis that estimates the effects of anti-HPV vaccination preventive strategies in Italy based on real-world data. Similar analyses have investigated only the burden of disease reduction attributable to HPV vaccine introduction according to the literature data [35, 36].

This work is one of the first analyses to evaluate the effects of anti-HPV interventions based on real-world data. It is hoped that these results can be reconfirmed over time through the continuous monitoring of hospitalisations for GWs of the vaccinated cohort but also confirmed for HPV-related diseases whose incidence occurs at an older age.

In light of the results, it can be concluded that not vaccinating against a preventable disease represents a cost. Failure to vaccinate leads to persistent avoidable costs due to avoidable hospitalisations and deaths. Prevention interventions also supported by their cost-effectiveness rationale should be offered in a timely and comprehensive way to the population to prove their positive economic impact.
